# The intestinal interferon system and specialized enterocytes as putative drivers of HIV latency

**DOI:** 10.3389/fimmu.2025.1589752

**Published:** 2025-05-14

**Authors:** Rachel L. Creighton, Sean M. Hughes, Florian Hladik, Germán G. Gornalusse

**Affiliations:** ^1^ Department of Obstetrics and Gynecology, School of Medicine, University of Washington, Seattle, WA, United States; ^2^ Vaccine and Infectious Disease Division, Fred Hutchinson Cancer Center, Seattle, WA, United States; ^3^ Department of Medicine, Division of Allergy and Infectious Diseases, University of Washington, Seattle, WA, United States; ^4^ Department of Global Health, Schools of Medicine and Public Health, University of Washington, Seattle, WA, United States

**Keywords:** HIV latency, interferon, enterocytes, microfold cell (M-cell), interferon stimulated gene (ISG)

## Abstract

The barrier to HIV cure is the HIV reservoir, which is composed of latently infected CD4^+^ T cells and myeloid cells that carry stably integrated and replication-competent provirus. The gastrointestinal tract (GIT) contains a substantial part of the HIV reservoir and its immunophysiology could be especially conducive for HIV persistence and reactivation. However, the exact cellular microenvironment and molecular mechanisms that govern the renewal of provirus-harboring cells and proviral reactivation in the GIT remain unclear. In this review, we outline the evidence supporting an overarching hypothesis that interferon activity driven by specialized enterocytes creates a microenvironment that fosters proliferation of latently infected CD4^+^ T cells and sporadic HIV reactivation from these cells. First, we describe unique immunologic features of the gastrointestinal associated lymphoid tissue (GALT), specifically highlighting IFN activity in specialized enterocytes and potential interactions between these cells and neighboring HIV susceptible cells. Then, we will describe dysregulation of IFN signaling in HIV infection and how IFN dysregulation in the GALT may contribute to the persistence and reactivation of the latent HIV reservoir. Finally, we will speculate on the clinical implications of this hypothesis for HIV cure strategies and outline the next steps.

## 1 Introduction

Except for a few isolated cases, HIV infection has never been cured (1). This is because HIV integrates into the host genome (becoming a “provirus”), evading the immune response and escaping antiretroviral therapies (ART) (2, 3). When ART is stopped, reactivation of proviruses in some latently infected cells leads to rebound viremia (4, 5). HIV latency is established very early during acute HIV infection, either through direct infection of resting memory CD4^+^ T cells or through infection of actively replicating CD4^+^ T cells that are later induced to a resting state (6–10). Latently infected cells are present in numerous microanatomical environments, including the blood, lymph nodes, brain, and gut (11–15). Tissue-specific factors like cell signaling, cell-cell interactions, and local antiretroviral drug concentration are critical to understanding the persistence and reactivation of latent HIV infection (14, 15).

**Figure 1:**
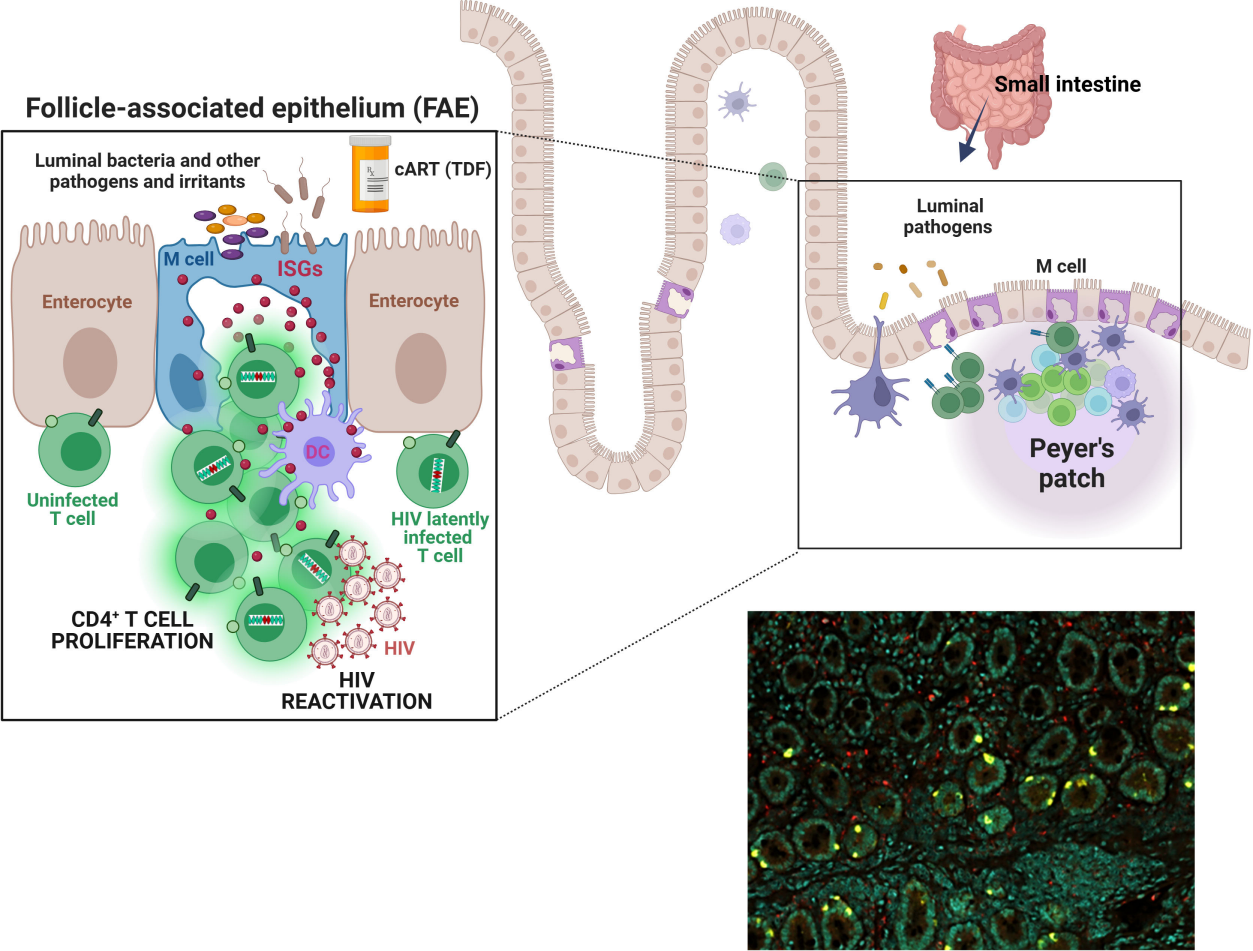
M cells or ISG^high^ enterocytes may foster HIV latency and reactivation. *Top schema.* The epithelium of the intestinal mucosa contains a population of cells expressing extremely high levels of type I/III interferon-stimulated genes (ISGs). Notably, this ISG expression appears independent of direct interferon (IFN) stimulation and also does not coincide with IFN expression by these cells. Co-expression of glycoprotein 2 suggests that many of these enterocytes are microfold cells (M cells). M cells are ~10% of all enterocytes; they are most numerous in the epithelium covering lymphoid follicles (the follicle-associated epithelium, “FAE”) and Peyer’s patches in the small intestine. They transport pathogens and other foreign materials from the lumen to underlying antigen-presenting cells (APCs) to initiate immune responses. They also influence APCs and lymphocytes via cytokines (e.g., IL-1, ISG15) and chemokines (e.g., CCL9 and CXCL16), as well as by expressing HLA-II molecules for direct interaction with T cells. M cells have a large basolateral invagination that amplifies the cell surface and usually contains B and/or T cells. While the significance of ISG15 and other M cell-associated ISGs for the HIV reservoir remains to be investigated, stimulation of type I/III IFN pathways has been shown to play an important role supporting HIV reservoir persistence, likely via T cell proliferation, and HIV-1 reactivation. Our overarching hypothesis is that immunologically hyperactive M cells in the gut create a microenvironment that fosters proliferation of latently infected bystander CD4^+^ T cells, sporadic HIV reactivation from these cells, or both. Thus, M cells and, more broadly, ISG^high^ enterocytes may play a role in HIV persistence and/or viral rebound post-ART cessation. *Bottom right image*. Immunostaining of a duodenal biopsy for ISG15 (ISG^high^ enterocytes, yellow) and CD68 (macrophages, red); DAPI signal denoted in teal. A formalin-fixed paraffin-embedded (FFPE) section was analyzed from a study participant that was exposed to PrEP (a combination of tenofovir disoproxil fumarate and emtricitabine) for two months (16).

Given its constant exposure to commensal bacteria and pathogens, the GIT is a highly immunologically active site. Previously, we found a population of cells in the intestinal epithelium producing extremely high levels of type I/III interferon (IFN)-stimulated proteins, including IFN-stimulated gene 15 (ISG15) (16). Co-expression of glycoprotein 2 suggests that some of these cells are microfold cells (M cells) (17). In response to viral pathogens, secreted IFNs upregulate the expression of interferon-stimulated genes (*ISGs*) to inhibit viral replication and prevent further cellular infection (18). However, in chronic HIV, the antiviral effect of the interferon system becomes pathological due to years of overstimulation (19, 20). This dysregulation of the IFN system, termed “interferonopathy,” has been posited to antagonize a potential HIV cure by driving bystander T cell proliferation, including of latently infected cells, thus contributing to HIV reservoir persistence (21–23). CD4+ T cell proliferation is thought to be the most important mechanism sustaining the HIV reservoir (24–38). There is also evidence that IFN efficiently reactivates HIV-1 (39). Therefore, the high IFN signaling activity observed in the intestinal epithelium led us to hypothesize that these immunologically active enterocytes play a role in the persistence and spontaneous reactivation of the HIV reservoir in the GIT (**Figure 1**).

## 2 The gastrointestinal tract, especially the gut-associated lymphoid tissue of the small intestinal tract, contains the largest HIV reservoir

Numerous studies in humans and non-human primates (NHP) have demonstrated that the largest HIV reservoir resides in the GALT. An analysis by Yukl et al. in people living with HIV (PLH) on ART estimated that 83-95% of HIV-infected cells reside in the GIT (40). Likewise, a survey in SIV-infected NHP on ART showed that ~98% of SIV vRNA^+^ (indicating active transcription and possibility for rebound) cells resided in the GIT (41). This continued low level production of SIV RNA, despite ART, also correlated with the presence of a large pool of SIV DNA^+^ cells (41). Studies in PLH on ART analyzing only the rectum have consistently found HIV DNA-containing cells (42, 43). The few studies of the upper GIT of PLH consistently identified the small intestinal tract as an important site for harboring HIV DNA^+^ and RNA^+^ cells (40, 44–51). HIV DNA (both clade B (46, 48–50) and C (47)) is present at higher levels in the small intestine than the blood. In addition, HIV RNA is more often found in the small intestine than the blood, and the RNA/DNA ratio is higher (40, 49). This site also has higher levels of activated CD4^+^ T cells than blood, which are suitable targets for infection (40, 49, 50). Thus, a large portion of the HIV reservoir resides in the GIT and HIV reactivation occurs at this site even during treatment.

Additional studies suggest that the HIV reservoir is further compartmentalized within the GIT, although there is some disagreement regarding which section of the GIT harbors the largest reservoir (44–46, 50–52). A recent study by Vellas et al. demonstrated an enrichment of intact proviruses in the ileum and colon compared to the duodenum of virally suppressed PLH (52). Another study identified increased HIV DNA concentrations along the GIT (40), while others found comparable levels in the ileum and rectum (45, 46, 50). With regards to viral transcription, studies agreed that higher levels of HIV RNA are present in the ileum compared to the rectum (40, 45). Further evidence of ongoing productive infection events in the small intestine during ART comes from an ART intensification study, where addition of raltegravir with or without a second antiretroviral drug caused a decrease of unspliced HIV RNA only in the ileum and not in other sites (peripheral blood, duodenum, colon, or rectum) (44). Overall, many studies point to the small intestine as an important and likely functionally unique HIV reservoir site, in particular as a hotspot for viral reactivation.

Several factors could explain the large latent HIV reservoir in the GIT compared to other anatomical sites. The GIT and GALT tissues are seeded rapidly and massively during the initial HIV infection phase, before ART is started (53–55). Compared to blood, a larger proportion of CD4^+^ T cells in the GIT express the HIV co-receptors CCR5 and CXCR4 and the gut homing receptor α4β7, making them highly susceptible to HIV infection (55–57). Furthermore, GALT contains many B cell follicles, which have been characterized as HIV “sanctuary” sites due to CD8^+^ T cell depletion, enabling continued productive infection of T follicular helper cells (58). Lastly, once ART is started, some areas of the GIT may experience incomplete tissue penetration of ART drugs (59, 60).

## 3 GALT immunological function

Here, we review GALT-specific cell types and immunological functions that may contribute to maintaining the HIV reservoir in the GIT. We specifically highlight microfold (M) cells, immunologically active cells that are especially enriched in the small intestine and interact closely with cell types known to harbor latent HIV.

GALT is distributed throughout the GIT and consists of multi-follicular structures (Peyer’s patches, cecal patches, colonic patches) and isolated lymphoid follicles (61–63). The immune structures and cell populations vary substantially along the length of the GIT (reviewed by Mowat et al (61)). Multi-follicular structures are most concentrated in the ileum and consist of germinal centers rich in naïve B cells and follicular dendritic cells surrounded by T cell-rich regions (61, 62, 64). Isolated lymphoid follicles have a similar structure as Peyer’s patches, but are much smaller (a single follicle compared to 10–100 follicles in Peyer’s patches) (63). Unlike multi-follicular structures, isolated lymphoid follicles are distributed along the entire length of the GIT, and their frequency increases 3 fold from the cecum to the rectum (65, 66). T cell populations in follicular structures include naïve CD4^+^ T cells, central memory CD4^+^ T cells, FOXP3^+^ regulatory T cells, and T follicular helper cells (62, 63).

While organized lymphoid structures are specialized for the generation of antigen-specific B cell responses, other immune cells distributed throughout the epithelium and lamina propria are specialized for effector responses and epithelial barrier homeostasis (62, 67–69). Intraepithelial lymphocytes (primarily CD8^+^) in the intestinal epithelium serve a wide variety of functions, including maintenance of the epithelial barrier, immune regulation, and antigen-specific cytotoxic effector responses (reviewed in (67)). The lamina propria contains CD4^+^ T cells with effector memory, transitional memory, Th17, and Th22 phenotypes, together with a variety of innate immune cells (62, 68, 69). These CD4^+^ T cells are of particular relevance due to their susceptibility to HIV infection and ability to harbor latent provirus. The differentiation of these and other immune cell types in the GIT are strongly influenced by dietary components like vitamin A and aryl hydrocarbon receptor ligands and by commensal microbiota and their metabolites (e.g., short chain fatty acids) (70–72).

The epithelium overlying GALT lymphoid follicles contains microfold cells (M cells), which are specialized for uptake and transport of luminal antigens (most eminently studied by Dr. Marian Neutra in the 90 and 00s) (17, 73–78). M cells contain a large basolateral invagination that enables close association with mononuclear phagocytes and intraepithelial B and T cells (73, 75). Antigens are sampled via endocytosis or pinocytosis and transported to the basolateral membrane in vesicles (17, 73, 74). M cells express cytokines (e.g., IL-1 (79), ISG15 (16)) and chemokines (e.g., CCL9 (80) and CXCL16 (81)) to recruit lymphocytes and leukocytes to the basolateral pocket. Some studies also suggest that M cells can express HLA-II molecules (82, 83) for direct interaction with T cells.

Conservatively estimated, under healthy conditions, there are 5×10^9^ M cells or M cell-like enterocytes in the human gut (84). Under pro-inflammatory conditions, such as infection or inflammatory bowel disease, their proportion can increase dramatically, either by trans-differentiation from mature enterocytes or *de novo* differentiation from crypt stem cells (though the exact mechanisms of M cell formation remain unknown) (73, 85–87). For example, *Salmonella typhimurium* infection causes increased density of M cells. The mechanism is thought to be via a bacterial effector protein activating RANKL expression in intestinal epithelial cells and inducing epithelial to mesenchymal transition (86).

Furthermore, M cell signaling is influenced by extracellular factors. In a study investigating the effects of two nucleoside/nucleotide reverse transcriptase inhibitors (NRTI)-class drugs in three clinical trials, we found that oral tenofovir disoproxil fumarate (TDF) and emtricitabine (FTC) taken as pre-exposure prophylaxis (PrEP) activated interferon pathways in the intestinal mucosa [**Figure 1** and (16)]. The most significantly upregulated genes were *IFI6* (IFN α-inducible protein 6), *ISG15* (Interferon-Stimulated Protein 15kDa), and *MX1* (MX dynamin-like GTPase 1). By co-staining with glycoprotein 2 (GP2) (17) we identified a portion of these cells as mature M cells (16). Similar ISG-expressing enterocytes have been described by others (88–90), with ISG expression modulated by inflammatory conditions like Crohn’s disease, ulcerative colitis, and environmental enteropathy (88, 89).

## 4 Dichotomous functions of IFN in HIV and the GIT

As indicated in the previous section, our study in people living without HIV demonstrated an immunostimulatory effect of the NRTIs TDF and FTC, which are commonly used as part of ART. Differential gene expression analysis revealed that 13 genes were significantly induced when comparing pretreatment baseline to 60 days of daily oral TDF/FTC PrEP by microarrays (16). Seven of these 13 genes (*IFI27*, *IFI6*, *IFIT1*, *ISG15*, *RSAD2*, *MX1*, and *OAS1*) are members of the Gene Ontology biological process “type I IFN signaling pathway”; four of the other 6 are known to be induced by type I IFN (*DDX60, SAMD9, IFI27L1* and *HERC6).* Thus, drugs from the NRTI class, which are mainstay ART components, may play a role in the persistent and largely unexplained immune activation seen in PLH whose HIV infection is otherwise well-controlled. In the paragraphs below, we discuss how IFNs, particularly type I (IFN-α and β) and type III (λ), can have contrasting functions, being protective during early events of viral infection and detrimental if their expression is dysregulated in chronic infection.

The antiviral activity of type I IFN is beneficial in acute HIV/SIV infection (91, 92). IFN-α2a administration during early SIV infection in rhesus macaques led to upregulation of ISGs (*MX1*, *MX2*, *OAS2*, *IRF7*) and a delay in systemic infection (93). Another study in rhesus macaques demonstrated that IL-21 therapy (known to induce NK cell proliferation and maturation (94)) followed by IFNα therapy resulted in a smaller SIV reservoir and delayed viral rebound during ART interruption (95). Meanwhile, in humans, delivery of pegylated IFN-α2b in combination with ART resulted in decreased GALT HIV RNA^+^ cells and blood HIV DNA^+^ cells. These changes correlated with increased GALT NK and T cell activation and upregulation of genes related to NK cell mediated immunity and IFN signaling (96, 97). In individuals coinfected with hepatitis C virus and HIV, immunotherapy with pegylated IFN-α2a further reduced proviral HIV DNA levels, which correlated with NK antiviral function (98–100). At the molecular level, type I IFN inhibits HIV-1 virus release through ISG15-mediated inhibition of ubiquitylation of the HIV-1 Gag protein (101), and it induces a number of directly-acting HIV restriction factors, e.g., MX1, TRIM5α, tetherin, and APOBEC3G (102–104).

In the context of chronic HIV infection, stimulation of type I/III IFN pathways can exacerbate the infection rather than clear it (105–107). Two recent studies in a humanized mouse model of chronic HIV infection showed that disrupting IFN-I/III pathways by blocking the IFN-α/β receptor 1 led to less immune activation, a lower HIV reservoir in lymphoid tissues, and delayed HIV rebound following ART interruption (21, 22). These studies were highlighted in a commentary by Deeks et al. titled “The interferon paradox: can inhibiting an antiviral mechanism advance an HIV cure?” (23). Likewise, in an NHP model of chronic ART-treated SIV infection, blockade of IFNα resulted in SIV reservoir reduction and better clinical outcomes during ART interruption (108). In PLH, chronic activation of IFN pathways has been associated with worse disease outcomes (20), partly driven by immune suppression and CD8^+^ T cell exhaustion and senescence (19, 107, 109). This dichotomous role of IFN in HIV pathogenesis is especially illustrated by studies comparing nonpathogenic to pathogenic SIV. Natural hosts like African Green Monkeys generate robust interferon responses, but the responses rapidly diminish following acute infection and these animals have minimal pathogenic sequelae (110). In contrast, chronic IFN activation occurs in animals like rhesus macaques, which have pathogenic SIV infection (111).

Several molecular mechanisms may underlie the deleterious effect of type I/III pathway activation on HIV persistence and reactivation. Type I IFN drives bystander T cell proliferation (112), which likely includes latently infected cells and thus may contribute to reservoir maintenance. It also facilitates the establishment of viral latency in monocyte-derived macrophages *in vitro* through the formation of inaccessible chromatin in the HIV provirus (113). In addition, IFN-α can reactivate HIV-1 from latently infected CD4^+^ T cells, potentially via STAT5 phosphorylation (39).

Most studies of IFN’s antiviral or deleterious effects have been performed with blood immune cells. However, the GIT is where preferential acute HIV-1/SIV replication, massive CD4^+^ T cell depletion, and microbial translocation occurs (53, 55), and where the largest HIV reservoir in the body resides (40, 41). In a study comparing long term non-progressors to people with high HIV viral loads, HIV-specific IFNγ secretion from GIT CD8+ and CD4+ lymphocytes was higher in the non-progressor group (114). This suggests a protective antiviral function of IFNγ in the GIT. During HIV/SIV infection, plasmacytoid dendritic cells (pDCs) upregulate β7-integrin expression, resulting in accumulation in the GIT (115, 116). pDCs produce large amounts of type I IFN during HIV/SIV infection, but this activity is reduced in natural SIV hosts (117). Blockade of α4β7 reduced the pDC population and immune activation in the colorectum of SIV-infected rhesus macaques (116). Together, these studies suggest that type I IFN produced by pDCs contributes to chronic immune activation in the GIT.

Dendritic cells and intra-epithelial CD45+ leukocytes also participate in type I/III IFN signaling in the GIT. In the absence of infection, commensal bacteria stimulate IFN secretion from these cells, which then leads to an ISG-mediated antiviral state in intestinal epithelial cells (118, 119). In acute HIV infection, CD4+ T cell depletion in the GIT leads to epithelial barrier dysfunction and microbial dysbiosis that persists even after stable ART (120–122). It is unknown how this persistent disruption of the gut epithelium affects IFN signaling, but in PLH on ART, gut ISG levels positively correlated with gut HIV-1 RNA and markers of immune activation, microbial translocation, and inflammation (124). In the next sections, we will speculate on the potential importance of the interaction between ISG^high^ enterocytes such as M cells and the HIV reservoir.

## 5 Potential role of ISG-expressing enterocytes in GALT HIV reservoir maintenance and rebound

**Figure 2:**
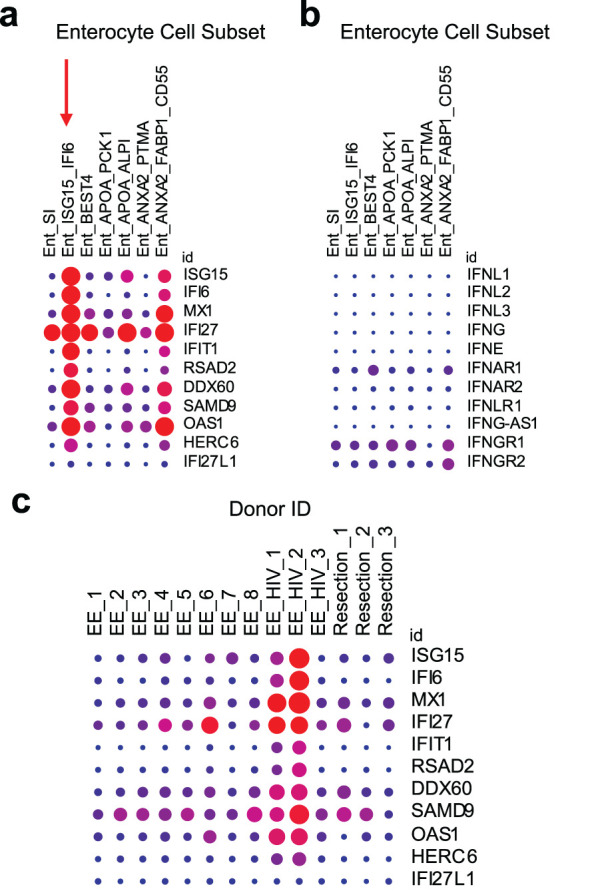
ISG expression is elevated in an enterocyte subset of the small intestine and in HIV infection. Kummerlowe et al (89) performed single cell RNA sequencing of small intestine biopsies from people with environmental enteropathy (EE) (including 3 adults living with HIV, EE_HIV) and people without environmental enteropathy (Resection). **(a)** Exploration of ISG expression in the data set revealed an enterocyte subset (Ent_ISG15_IFI6) with high ISG expression (denoted with a vertical red arrow), but **(b)** little or no IFN or IFN receptor expression. **(c)** A similar pattern of high ISG expression was identified in study participants living with HIV. The data was accessed and the figure was generated using the Single Cell Portal from the Broad Institute (170). In all plots, the dot size represents the percent of the cell population expressing the gene, and the color represents the scaled mean expression from 0 (blue) to 1 (red) across all cell subsets **(a, b)** or all study participants **(c)**. These plots were generated automatically after searching for the ISGs of interest. Experimental and data analysis details are available in the original paper (89).

The effect of HIV infection on IFN-signaling in GIT enterocytes is unknown, however GIT enterocyte ISG expression has been shown to be increased by small molecule drugs or autoimmune disease. As mentioned in Section 4, we observed an increase in the number of rectal and duodenal enterocytes expressing ISGs after 2 months of TDF/FTC PrEP (16). *In vitro* experiments have also demonstrated that nucleotide analogues can stimulate dose-dependent secretion of type III IFN (IFN-λ3) in GIT epithelial cells (123). To further validate the potential for ISG upregulation in GIT enterocytes, we explored scRNA-Seq datasets from Kummerlowe et al (89) and Smillie et al *(* 90 *)*, in the Broad Institute Single Cell Portal. These datasets were identified based on species (Homo sapiens), organ (gastrointestinal tract), cells (microfold cells), and genes (ISGs from (16)) of interest. In both datasets, we identified a subset of enterocytes in the duodenum that co-express high amounts of ISGs (*ISG15*, *IFI27*, *IFI6*, *MX1, IFIT1*, etc.) (**Figure 2a**, denoted with a red arrow) but, notably, not interferons or interferon receptors (*IFNL1, IFNL2, IFNL3, IFNG, IFNE, IFN-alpha receptor 1 or 2, or IFN-lambda receptor IFNLR1*) (**Figure 2b**). Results from these studies align well with our tenofovir study (16), in which type I IFNs (IFN-α and -β) and type III IFNs (IFN-λ1–4) were not detectable. In Kummerlowe et al (89) and Smillie et al *(* 90 *)*, the ISG^high^ subset of cells did not express GP2, suggesting they are not mature M cells but another type of enterocyte (17). Although both studies included samples from participants with gastrointestinal disease, expression of the same ISGs in enterocytes from our TDF/FTC PrEP study suggests that this pattern of ISG expression is not specific to gastrointestinal disease and that further study of enterocyte ISG expression in HIV is warranted.

The Kummerlowe study also showed an overall increase in ISG expression (**Figure 2c**) and specifically an increased fractional abundance of the ISG^high^ enterocyte subset in ART-treated HIV infection (Supplementary Figure S7 in Kummerlowe et al (89)). The increase in this ISG^high^ enterocyte population during chronic HIV infection suggests a role for these cells in HIV persistence and reactivation. It is unknown how HIV-infected cells respond to M cell-derived ISGs and if this interaction can drive HIV reservoir persistence and/or reactivation. Briefly, we will discuss potential effects of ISGs on the HIV reservoir in the GIT using ISG15 as an example.

### 5.1 ISG15: an M cell-derived ISG with the potential to enhance latent HIV-infected cell proliferation and HIV reactivation

ISG15 is one of the most strongly (125) and rapidly induced (126) ISGs. It has both intracellular innate immune and secreted cytokine-like functions (127, 128). ISG15 is a member of the ubiquitin family and is induced by viral and bacterial infections (129–131), and also directly by IFNs (132, 133). Intracellularly, it binds covalently to target proteins through a process called ISGylation, which serves a key role in innate immunity, specifically inhibiting viral infection and viral release (128). As a cytokine, ISG15 induces lymphocyte proliferation, IFN-γ production, and neutrophil chemotaxis (134, 135). Soluble ISG15 can also stimulate a strong release of pro-inflammatory cytokines such as IL-6, TNF-α, and IL1β (88). These effects are triggered by ISG15 binding the integrin receptor lymphocyte function-associated antigen 1 (LFA1) on NK cells and T cells (136). The specific functions of ISG15 in the GALT and in M cells are unknown.

Regarding HIV-1 pathogenesis, several effects of ISG15 have been reported. ISG15 is upregulated in dendritic cells and macrophages in response to HIV-1 provirus (137), and in PLH, ISG15 mRNA levels in PBMCs correlated with HIV-1 viral load and markers of worse disease outcome (138). ISG15 inhibits HIV-1 virus release by inhibiting ubiquitination of the HIV-1 Gag protein (101). Conversely, intracellular ISG15 was also shown to increase HIV-1 replication in primary CD4^+^ T cells (139). This could occur via ISG15-mediated stabilization of USP18, a negative regulator of JAK-STAT signaling (140, 141). Overall, despite ISG15 now being intently studied, there is still relatively little known regarding its role in the GIT, HIV infection, and HIV latency.

Taken together, ISG15 expression by enterocytes may affect HIV persistence and reactivation in two ways. First, enterocyte-secreted ISG15 may promote proliferation of neighboring T cells, some of which could be latently infected, thus maintaining or growing the size of the reservoir. It may also recruit and activate CD4^+^ T cells, which are targets for infection. Second, ISG15 may play a role in viral reactivation of latently infected T cells by stimulating the release of pro-inflammatory cytokines, which then reactivate HIV. Thus, despite its direct intracellular inhibitory effects on HIV, ISG15 expression by enterocytes may drive persistence of the latent HIV reservoir as well as viral reactivation from T cells. Similar effects are likely to result from other ISGs produced by enterocytes.

### 5.2 IFN-independent induction of ISGs

As mentioned above, ISG^high^ enterocytes appear to express few IFN receptors, which suggests that their ISG expression may be independent of IFN stimulation. Transcriptional regulation of ISGs can be activated by IFNs via the classical JAK-STAT pathway or through non-canonical IFN-independent pathways (reviewed in (142)). These non-canonical signaling pathways, including the activation of mitogen-activated protein kinases (MAPKs), mammalian target of rapamycin (mTOR), protein kinase C (PKC), IRF3, or NF-κB, can be activated by cellular stress (e.g., heat shock, DNA damage, oxidative stress) or viral infections (18, 143).

A recent study suggested that ISG induction occurs via NF-κB signaling in an enteroid model of M cells. Ding et al. developed a culture system to generate glycoprotein 2 (GP2) positive M cells in human ileal enteroids using a variety of differentiation factors (RANKL, retinoic acid, and lymphotoxin α_2_β_1_) (144). Transcriptomic analysis showed that this lymphotoxin-mediated, IFN protein-independent signaling induced upregulation of several ISGs (*IFI6, IFI44, IFITM1, IFIT1, RSAD2*) in enteroids with induced M cells (144). This ISG expression prevented rotavirus infection specifically in GP2-positive M cells in the enteroid model (144).

A second IFN-independent mechanism of ISG induction occurs via pattern recognition receptors (PRRs). M cells express PRRs such as Toll-like receptors (TLRs) (145) and nucleotide-binding oligomerization domain-containing proteins (NODs) (146). These receptors recognize microbial molecular motifs, and can trigger the activation of signaling pathways that converge in ISG induction without the need for IFNs. The TLR3 ligand poly(I:C) induced expression of pSTAT1, IRF9, and free ISG15 independently of autocrine or paracrine IFN signaling in an organoid model (88). ISG15 is induced directly by HIV-1 provirus in CD4^+^ T cells, macrophages, and dendritic cells via MDA5 (147), a RIG-I like receptor that can be expressed by enterocytes (89).

These pathways could be involved in the apparent constitutive ISG expression we observed in enterocytes (16), given their constant exposure to and sampling of the intestinal lumen.

As a caveat, while the ISG-expressing subset of enterocytes did not strongly express IFN or IFN receptors in the studies described at the beginning of Section 5, there were other enterocyte subsets expressing IFN-α receptor 1 and IFN-γ receptors 1 and 2 (89, 90). Additionally, the absence of type I/III IFNs in our tenofovir study (16) could be due to the low sensitivity of the microarray used. Therefore, these previous studies do not exclude the possibility that IFN is involved upstream of the ISG expressing enterocytes.

## 6 Clinical implications for HIV cure strategies

Understanding how M cells affect the HIV reservoir may enable us to improve experimental HIV cure interventions. Two prominent approaches to curing HIV are “shock and kill” (“sterilizing cure” (148)) and “block and lock” (“functional cure”) (149, 150). “Shock and kill” (also named “kill and kill” or “activation-elimination”) induces HIV reactivation with cytokines and latency reversing agents (LRAs). These LRAs include protein kinase C (PKC) modulators, mitochondrial-derived activators of caspases (SMAC) mimetics, BET-bromodomain inhibitors, histone deacetylase (HDAC) inhibitors, and others (reviewed in (151)). In theory, reactivated cells should be eliminated by viral cytopathic effects or the immune system, but *in vitro* data and clinical trials have shown that latency reversal alone does not effectively decrease the size of the HIV reservoir (152–155). A combination of LRAs may be more effective (156–158), but such approaches can be toxic. It is possible that studying the immune modulatory effects of ISG^high^ enterocytes and M cells could reveal novel and less toxic approaches for latency reversal.

The “block and lock” functional cure strategy (149) is based on inducing deep latency in the HIV reservoir by using latency-promoting agents (LPAs) such as cortistatin A (159, 160), Janus kinase (Jak)-STAT inhibitors (161), and bromodomain-containing protein 4 (BRD4) modulators (162). This approach aims to permanently silence all latent proviruses, preventing the transcription of replication-competent proviruses and blocking actively replicating viruses. Thus, LPAs could maintain functional cure following ART interruption (159, 163). It remains unknown whether immune activation, e.g., by other infections, antagonizes this strategy. Further studies of ISG^high^ enterocytes like M cells could be critical to define whether LPAs can overcome endogenous signals that trigger HIV reactivation.

## 7 Conclusions and next steps

In summary, in this review we argue that HIV reservoir persistence and reactivation in the gut, especially the small intestine, is mediated by ISG expression in M cells or M cell-like cells. Our argument is based on three key facts (1): the GIT contains the majority of the cells in the HIV reservoir (40, 41); (2) microfold (M) cells are uniquely enriched in the mucosa of the small intestine, interact closely with T cells and other mucosal leukocytes (73–75), and express extremely high levels of ISGs (16, 88–90); and (3) IFN signaling can enhance T cell proliferation and HIV reactivation (21, 22, 39, 112). Thus, M cells, or broadly ISG^high^ enterocytes, may foster a microenvironment that is especially conducive to maintaining the latent HIV reservoir and/or allowing HIV reactivation in adjacent HIV-infected T cells or macrophages.

M cells are key to the immune environment of the gut. However, their isolation and *ex vivo* culture is challenging, and there are no available immortalized M-cell lines. Animal models are also of limited utility because M cells are highly variable across species (164, 165). Single-cell transcriptomic data from M cells have been collected from dissociated tissues (89, 90, 166, 167), confirming their high ISG expression, but not yet within their spatial context *in situ*. Similarly, the effects of M cells on neighboring immune cells in the GALT have not yet been studied because until recently the respective methods had not been available. Today, with the advent of single-cell spatial multiomics *in situ (* 168, 169 *)*, this limitation has been overcome. Jointly mapping genomic, epigenomic, transcriptomic, proteomic and metabolic profiles from single cells in their spatial context will shed light on these specialized enterocytes in health and disease. Spatial analyses will be able to address very specific functional questions, namely how T cells and macrophages respond to the influence of neighboring M cells. This may lead to a deeper understanding of HIV latency in the gut, as well as, more broadly, the pathogenesis of enteric infections and autoimmune disorders, and the design of oral vaccines.
